# Detection of Heteromers Formed by Cannabinoid CB_1_, Dopamine D_2_, and Adenosine A_2A_ G-Protein-Coupled Receptors by Combining Bimolecular Fluorescence Complementation and Bioluminescence Energy Transfer

**DOI:** 10.1100/tsw.2008.136

**Published:** 2008-10-11

**Authors:** Gemma Navarro, Paulina Carriba, Jorge Gandí, Francisco Ciruela, Vicent Casadó, Antoni Cortés, Josefa Mallol, Enric I. Canela, Carmen Lluis, Rafael Franco

**Affiliations:** ^1^ Institut d'Investigacions Biomèques August Pi i Sunyer Centro de Investigación Biomédica en Red Sobre Enfermedades Neurodegenerativas (CIBERNED), y Departamento de Bioquímica y Biología Molecular, Facultad de Biología, Universitat de Barcelona, Spain; ^2^ Unitat de Farmacologia, Departamento Patologia i Terapèica Experimental, Facultat de Medicina, Universitat de Barcelona, L'Hospitalet de Llobregat, Spain; ^3^ CIMA Neurociencias, Universidad de Navarra, Pamplona, Spain

**Keywords:** G-protein-coupled receptors, adenosine A_2A_ receptor, cannabinoid CB_1_ receptor, dopamine D_2_ receptor, heterotrimers, oligomers of three different protomers, bimolecular fluorescence complementation, bioluminescence energy transfer, BRET

## Abstract

Functional interactions in signaling occur between dopamine D_2_ (D_2_R) and cannabinoid CB_1_ (CB_1_R) receptors, between CB_1_R and adenosine A_2A_ (A_2A_R) receptors, and between D_2_R and A_2A_R. Furthermore, direct molecular interactions have been reported for the pairs CB_1_R-D_2_R, A_2A_R-D_2_R, and CB_1_R-A_2A_R. Here a combination of bimolecular fluorescence complementation and bioluminescence energy transfer techniques was used to identify the occurrence of D_2_R-CB_1_R-A_2A_R hetero-oligomers in living cells.

## INTRODUCTION

The mechanism of action responsible for the motor-depressant effects of cannabinoids, which operate through centrally expressed cannabinoid $CB_1$ receptors ($CB_1R$), is still a matter of debate. The cellular and subcellular localization of $CB_1R$ and $D_2$ receptors ($D_2R$) in the basal ganglia have been described in detail[1,2,3,4,5]. The evidence suggests a colocalization of striatal $CB_1R$ and $D_2R$ predominantly in the soma and dendrites of the GABA enkephalinergic neurons and also in corticostriatal glutamate terminals where $A_{2A}$ receptors ($A_{2A}R$) are also present[5,6,7,8,9,10].

Functional interactions between $CB_1R$ and $A_{2A}R$ or $D_2R$ receptors that are relevant for striatal function have been reported. Thus, $CB_1R$ and $A_{2A}R$ form heteromers in cotransfected HEK-293T cells and in rat striatum[11]. In a human neuroblastoma cell line, $CB_1R$ signaling was found to be completely dependent on $A_{2A}R$ activation. Accordingly, blockade of $A_{2A}R$ counteracted the motor-depressant effects produced by the intrastriatal administration of a $CB_1R$ agonist[11]. The existence of $CB_1R$-$D_2R$ heteromers has been demonstrated in transfected cell lines by coimmunoprecipitation[12] and by fluorescence resonance energy transfer (FRET) experiments[13]. Antagonistic $CB_1R$-$D_2R$ interactions have been discovered at the behavioral level[13,14,15,16]. In rats, the $CB_1R$ receptor agonist CP 55,940 at a dose that did not change basal locomotion was able to block quinpirole-induced increases in locomotor activity. In addition, not only the $CB_1R$ antagonist rimonabant, but also the specific $A_{2A}R$ antagonist MSX-3, blocked the inhibitory effect of $CB_1R$ agonist on $D_2$-like receptor agonist-induced hyperlocomotion[13]. Taken together, these results give evidence for the existence of antagonistic $CB_1$-$D_2$ receptor-receptor interactions within $CB_1R$-$D_2R$ heteromers in which $A_{2A}R$ may also participate.

$A_{2A}R$-$D_2R$ was one of the first reported heteromers[17]. A close physical interaction between both receptors has been shown using coimmunoprecipitation and colocalization assays[17], and FRET and bioluminescence resonance energy transfer (BRET) techniques[18,19,20]. At the biochemical level, two kinds of antagonistic $A_{2A}R$-$D_2R$ interactions have been discovered that can explain the $A_{2A}R$-$D_2R$ interaction observed at both the functional and behavioral levels[9,21,22,23]. First, by means of an intramembrane interaction, i.e., by intramolecular cross-talk within the heterodimer, stimulation of $A_{2A}R$ decreases the affinity of $D_2R$ for their agonists[24]. Second, the stimulation of $D_2R$, a Gi/o protein–coupled receptor, inhibits the cAMP accumulation induced by the stimulation of the Gs/olf protein–coupled $A_{2A}R$[17]. Therefore, it has been suggested that the $A_{2A}R$-$D_2R$ interaction cross-talk in the central nervous system may provide new therapeutic approaches for Parkinson's disease, schizophrenia, and drug addiction[23,25].

Since trimers formed by cannabinoid, adenosine, and dopamine receptors were suspected, strategies to detect them were developed in our laboratory. In one of them, trimers were detected by sequential application of BRET and FRET[26]. In this paper, the formation of hetero-oligomer complexes formed by cannabinoid $CB_1$, dopamine $D_2$, and adenosine $A_{2A}$ G-protein-coupled receptors (GPCRs) is confirmed by another technique[27], which consists of combining bimolecular fluorescence complementation and bioluminescence energy transfer.

## MATERIALS AND METHODS

### Cell Culture

HEK-293T cells were grown in Dulbecco's modified Eagle's medium (DMEM) (Gibco) supplemented with 2 m*M* L-glutamine, 100 U/ml penicillin/streptomycin, and 5% (v/v) heat inactivated fetal bovine serum (FBS) (all supplements were from Invitrogen, Paisley, Scotland, U.K.). Cells were maintained at 37ºC in an atmosphere of 5% $CO_2$, and were passaged when they were 80–90% confluent, i.e., approximately twice a week.

### Fusion Proteins and Expression Vectors

Full-length YFP was subcloned in the XhoI site of pcDNA3.1 vector (Invitrogen). The N-terminal truncated version of YFP, named nYFP (amino acids 1 to 155), was made by PCR amplification and cloning into the XhoI site of pcDNA3.1 using the following primers: FnYFP (5'-

CCGCTCGAGACCATGGTGAGCAAGGGCGAGGAGC-3’) and RnYFP (5’-CCGTCTAGATCAGGCCATGATATAGACGTTG-3’). Also, a C-terminal truncated version of YFP, named cYFP (amino acids 155 to 231), was made using the same strategy and the following primers: FcYFP (5’-CCGCTCGAGACCATGGACAAGCAGAAGAACGGC-3’) and RcYFP (5’-CCGTCTAGATTACTTGTACAGCTCGTCCAT-3’). $G_{\alpha s}$ cloned in SFV1 vector (generously given by H. Vogel, Ecole Politecnique Federal de Laussane, Switzerland) or $G_\gamma$ and $G_\beta$ cloned in pEYFP-C1 vector (generously provided by S. Cotecchia, Department of Pharmacology and Toxicology, University of Lausanne, Switzerland) were amplified to miss their stop codons using sense and antisense primers harboring unique NheI and BamHI sites to clone $G_\alpha$ and $G_\beta$ in pcDNA3.1-nYFP and pcDNA3.1-cYFP, respectively, and HindIII and BamHI sites to clone $G_\gamma$ in *Rluc* vector (p*Rluc*-N1 PerkinElmer, Wellesley, MA). The amplified fragments were subcloned to be in-frame with the multiple cloning site of the vectors to give the plasmids $G_\alpha$-nYFP, $G_\beta$-cYFP, and $G_\gamma$-*Rluc*, respectively. All plasmids express luminescent or part of fluorescent proteins on the C-terminal ends of proteins. The human cDNAs for $A_{2A}R$, $CB_1R$, $D_2R$, or $D_{4.4}R$ cloned in pcDNA3.1 were amplified without their stop codons using sense and antisense primers harboring unique NheI and BamHI sites to clone $A_{2A}R$, $CB_1R$, and $D_2R$ in pcDNA3.1-cYFP, pcDNA3.1-nYFP, and p*Rluc*-N1, respectively, and XhoI and BamHI sites to clone $D_{4.4}R$ in p*Rluc*-N1 vector. The amplified fragments were subcloned to be in-frame with the multiple cloning site of the vectors to give the plasmids $A_{2A}$-cYFP, $CB_1$-nYFP, $D_2$-*Rluc*, or $D_4$-*Rluc*.

## Transient Transfection and Protein Determination

HEK-293T cells growing in six-well dishes were transiently transfected with the corresponding fusion protein cDNAs by PEI (PolyEthylenImine, Sigma, Steinheim, Germany) method. Cells were incubated with the corresponding cDNA, 5.47 m*M* (in nitrogen residues) PEI, and 150 m*M* NaCl in a serum-free medium. After 4 h, cells were placed in a fresh complete culture medium. Forty-eight hours after transfection, cells were rapidly washed twice in HBSS containing 10 m*M* glucose, detached, and resuspended in the same buffer. To control for cell number, sample protein concentration was determined using a Bradford assay kit (Bio-Rad, Munich, Germany) using bovine serum albumin as reference. Cell suspension (20 µg of protein) was distributed into 96-well black plates with a transparent bottom for fluorescence determinations or white plates with white bottom for BRET experiments.

## Fluorescence Measurements

To quantify fluorescence, cells (20 µg protein) were distributed in 96-well microplates (black plates with a transparent bottom) and fluorescence was read in a Mithras LB 940 (Berthold Technologies, DLReady, Germany) using a 10-nm bandwidth excitation and emission filters at 485 and 530 nm, respectively. Protein fluorescence expression was determined as fluorescence of the sample minus the fluorescence of cells that were not transfected.

## BRET Assays with Bimolecular Fluorescence Complemented Proteins

HEK-293T cells were transiently cotransfected with a constant amount of cDNA encoding for the protein fused to *Rluc* and with increasingly equal amounts of cDNA corresponding to proteins fused to one of the two complementary parts of the YFP protein (nYFP and cYFP). Fluorescence was measured as indicated above. The equivalent to 20 µg of cell suspension was distributed in 96-well microplates (Corning 3600, white plates with white bottom) and 5 µ*M* coelenterazine H (Molecular Probes, Eugene, OR) was added. After 1-min delay, collection of readings started using a Mithras LB 940, which allows the integration of the signals detected in the short-wavelength filter at 485 nm (440–500 nm) and the long-wavelength filter at 530 nm (510–590 nm). To quantify for protein-*Rluc* expression, luminescence readings were performed after 10 min of adding 5 μ*M* coelenterazine H. The net BRET is defined as [(long-wavelength emission)/(short-wavelength emission)]-Cf, where Cf corresponds to [(long-wavelength emission)/(short-wavelength emission)] for the *Rluc* construct expressed alone in the same experiment.

## Immunostaining

For immunocytochemistry, transiently transfected HEK-293T cells were fixed in 4% paraformaldehyde for 15 min and washed with PBS containing 20 m*M* glycine (buffer A) to quench the aldehyde groups. Then, after permeabilization with buffer A containing 0.2% Triton X-100 for 5 min, cells were treated with PBS containing 1% bovine serum albumin. After 1 h at room temperature, cells expressing $D_2$R-*Rluc* were labeled with the primary mouse monoclonal anti-*Rluc* antibody (1/100, Chemicon) for 1 h, washed, and stained with the secondary antibody Cy3 Donkey antimouse (1/100, Jackson Immunoresearch Laboratories, Baltimore, PA). Heterodimers of receptors fused to complementary fragments of YFP were detected by their fluorescence properties. Samples were rinsed and observed in a Leica SP2 confocal microscope (Leica Microsystems, Mannheim, Germany).

## RESULTS AND DISCUSSION

In the early 1980s, and based on indirect functional evidence, it was proposed that GPCRs could interact at the level of the neuronal plasma membrane. In the early 1990s, electrophoretic mobility and coimmunoprecipitation assays gave the first indication of GPCR homomerization. More recently, the development of the biophysical techniques BRET and FRET allowed the demonstration of GPCR homodimerization and heteromerization of two GPCRs in living cells[23,28,29,30,31,32,33,34,35,36]. Nevertheless, the lack of assays monitoring interactions between more than two proteins simultaneously makes it very difficult to draw a map of molecular networks involving protein-protein interactions. By the approach previously reported by Héroux et al.[27], we here show that the combination of bimolecular fluorescence complementation and bioluminescence energy transfer is useful to detect heteromerization of three different GPCRs.

For bimolecular fluorescence complementation assay, the reconstitution of a reporter fluorescence protein (YFP) from its two fragments attached to the potential interacting protein partners under study[37,38] is taken as evidence for the molecular interaction between the partners[39]. The usefulness of the bimolecular fluorescence complementation technique to detect protein heterodimers was proved using cells transfected with the following subunits of heterotrimeric G proteins: $G_\alpha$nYFP and $G_\beta$cYFP. Positive complementation was detected by the increase of fluorescence at 530 nm upon increasing the amount of transfected proteins (Fig. 1). As negative control, no signal was detected when either $G_\alpha$nYFP and cYFP, or $G_\beta$cYFP and nYFP, were cotransfected (Fig. 1). These results prove the ability of the bimolecular complementation technique to detect heterodimers. The bimolecular fluorescence complementation technique was used to detect $CB_1R$-$A_{2A}R$ heterodimers in HEK-293 cells. Fusion of nYFP and cYFP fragments to $CB_1R$ or to $A_{2A}R$ did not prevent the receptor functionality determined as ERK1/2 phosphorylation (results not shown). Cells transfected with $CB_1$RnYFP and $A_{2A}$RcYFP (see Methods) showed fluorescence at the membrane level (Fig. 2A). No fluorescence was detected when cells were cotransfected with $A_{2A}$RcYFP and nYFP (Fig. 2A) or with $CB_1$RnYFP and cYFP (results not shown), showing the specificity of the signal. In agreement, positive complementation was detected by the increase of fluorescence at 530 nm upon increasing the amount of transfected receptors (Fig. 2B). As a negative control, no signal was detected when either $A_{2A}$RcYFP and nYFP, or $CB_1$RnYFP and cYFP, were cotransfected (Fig. 2B). Taken together, these results validate the usefulness of the bimolecular fluorescence complementation technique to monitor the formation of $CB_1R$-$A_{2A}R$ heteromers.

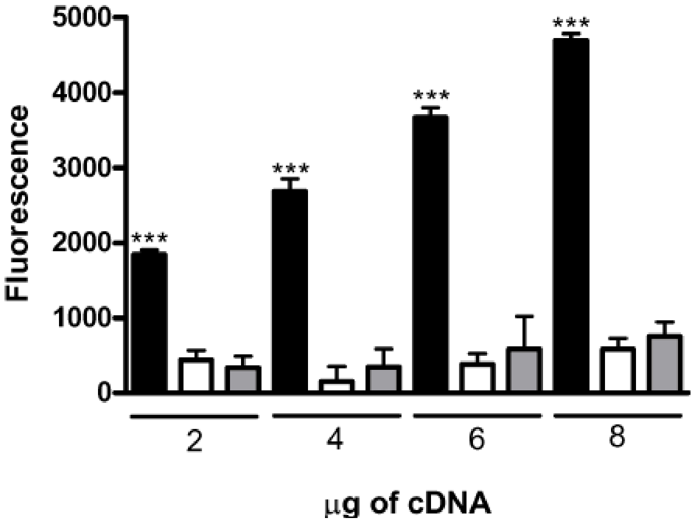

**FIGURE 1.** Molecular interaction between $G_\alpha$ and $G_\beta$ subunits of heterotrimeric G proteins detected by bimolecular fluorescence complementation. HEK-293 cells were cotransfected with equal amounts of cDNAs corresponding to the fusion proteins $G_\alpha$nYFP and $G_\beta$cYFP (black), $G_\alpha$nYFP and cYFP (white), or $G_\beta$cYFP and nYFP (grey). Forty-eight hours post-transfection, fluorescence was determined at 530 nm. Values are mean ± SEM of four independent experiments. One-way ANOVA followed by Newman-Keuls test showed significant differences respective to both negative controls. ***$p < 0.001$.

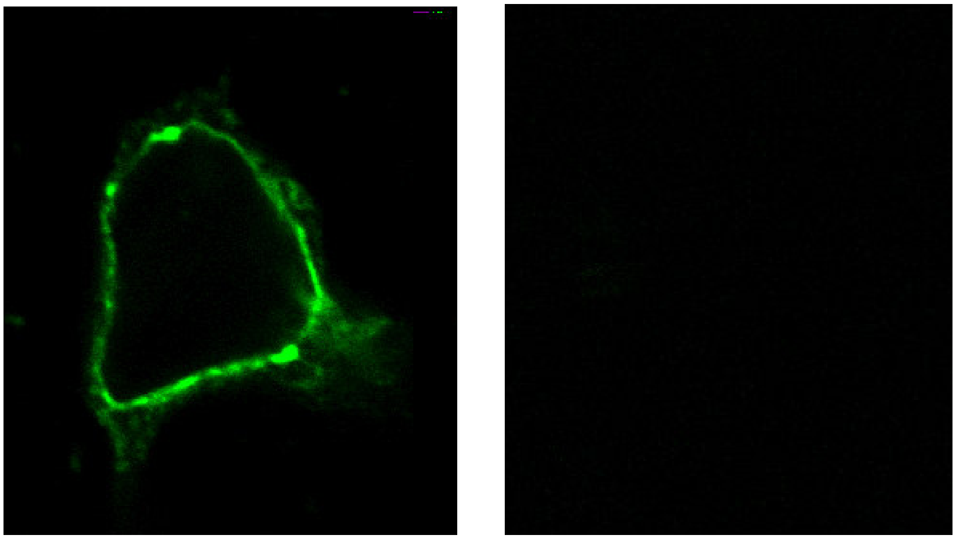

A

**FIGURE 2.** $CB_1R$-$A_{2A}R$ heterodimers in HEK-293 cells. HEK-293 cells were cotransfected with (A) 2 μg of cDNA corresponding to the fusion proteins $CB_1$RnYFP and $A_{2A}$RcYFP (left panel), or $A_{2A}$RcYFP and nYFP (right panel) or (B) equal amounts of cDNAs corresponding to $CB_1$RnYFP and $A_{2A}$RcYFP (black), $A_{2A}$RcYFP and nYFP (grey), or $CB_1$RnYFP and cYFP (white). In (A), confocal microscopy images obtained 48 h post-transfection are shown. In (B), fluorescence at 530 nm was determined 48 h post-transfection. Values are mean ± SEM of four independent experiments. One-way ANOVA followed by Newman-Keuls test showed significant differences respective to both negative controls. ***$p < 0.001$.

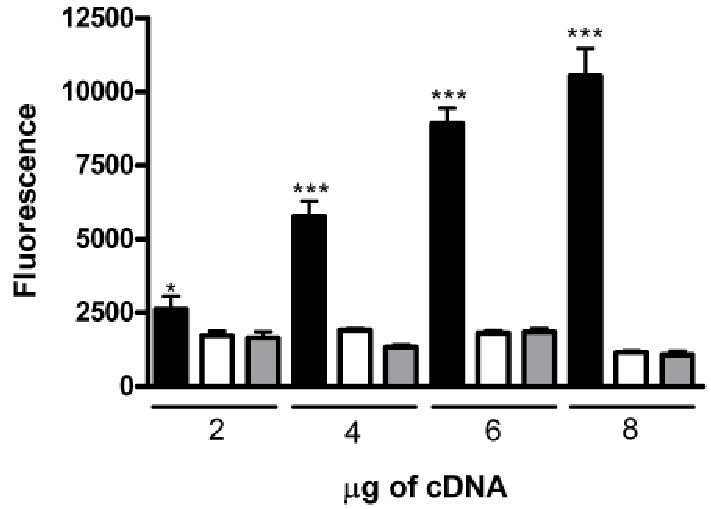

**FIGURE 2B**

The positive results on the formation of $CB_1R$-$A_{2A}R$ heteromers, obtained by the bimolecular fluorescence complementation technique, opened the possibility of combining this technology with BRET to investigate the existence of $D_2R$-$CB_1R$-$A_{2A}R$ hetero-oligomers. The usefulness of the combination of the bimolecular fluorescence complementation technique and BRET to detect oligomers formed by three different proteins was first tested by transfecting the following fusion proteins of the three subunits of heterotrimeric G proteins: $G_\gamma$-*Rluc*, $G_\alpha$nYFP, and $G_\beta$cYFP. Positive BRET was detected between $G_\gamma$-*Rluc* and complemented $G_\alpha$nYFP-$G_\beta$cYFP. A hyperbolic BRET saturation curve was obtained upon increasing the $G_\alpha$nYFP-$G_\beta$cYFP expression (Fig. 3A). This result proves the ability of the combination of the two techniques to detect trimolecular protein complexes as those detected for functional calcitonin gene-related peptide receptors, which are formed by the asymmetric assembly of a calcitonin receptor-like receptor homo-oligomer and a monomer of receptor activity-modifying protein-1[27].

In order to detect possible formation of hetero-oligomers composed of $CB_1$, $D_2$, and $A_{2A}$ receptors, HEK-293 cells were transfected with $D_2R$*Rluc*, $A_{2A}$RcYFP, and $CB_1$RnYFP. Fusion of *Rluc* to $D_2R$ did not prevent the receptor functionality determined as ERK1/2 phosphorylation (results not shown). Fusion proteins did not affect the normal subcellular distribution of receptors (Fig. 3B). In fact, these receptors are predominantly colocalized in the plasma membrane of cotransfected cells. In conditions to give a $BRET_{50}$ fusion protein expression levels, measured as described previously[26] by radioligand binding in different experimental sessions, were between 0.5 and 0.7 pmols/mg protein for $A_{2A}$RcYFP, between 0.9 and 1.1 pmols/mg protein for $D_2R$*Rluc*, and between 0.6 and 0.8 pmols/mg protein for $CB_1$RnYFP. Triggering with coelenterazin H, these transfected cells gave a significant BRET signal. The BRET signal was specific as assessed by the saturation hyperbola obtained upon increasing the complemented $A_{2A}$RcYFP-$CB_1$RnYFP expression and by the lack of the signal using $D_4$*Rluc* instead of $D_2$*Rluc* as a negative control (Fig. 3C). These data indicate that $D_2R$, $CB_1R$, and $A_{2A}R$ form, at least, trimolecular oligomers in cotransfected living cells. This technique is validated by the identification of $D_2$-$CB_1$- and $A_{2A}$ receptor heteromers by sequential resonance energy transfer (SRET)[26]. Apart from transmembrane regions, basic and acidic residues are involved in the epitope-epitope electrostatic interactions existing in $D_2$-$A_{2A}$ receptor heteromers. In fact, mass spectometry and pull-down assays have been instrumental to show that the Arg-rich $D_2R$ epitope may bind to two different epitopes in the C-terminal part of the $A_{2A}R$, one containing two adjacent Asp residues and another containing a phosphorylated Ser residue[18,20]. Then, it might be possible that one of the epitopes is involved in the interaction with $D_2$ and another in the interaction with $CB_1$. Further experimental work is necessary, however, to elucidate the amino acids constituting the interfaces in the $D_2$-$CB_1$-$A_{2A}$ hetero-oligomer.

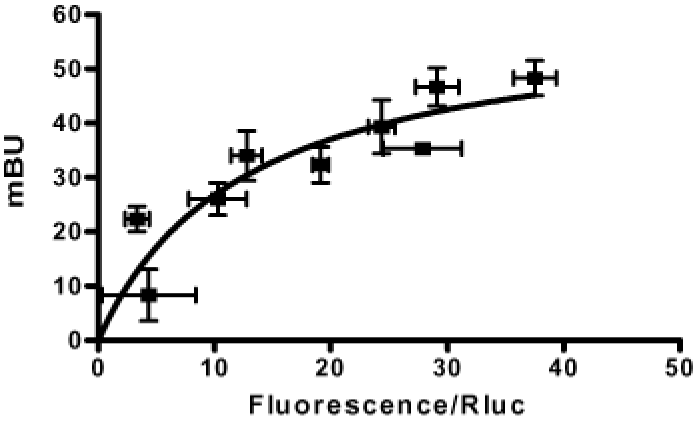

A

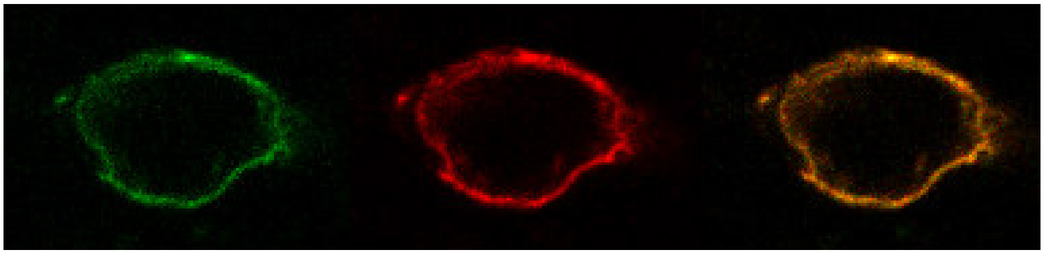

B

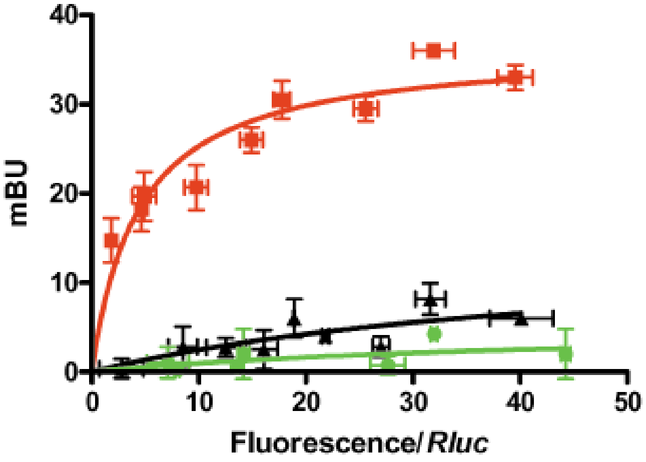

C

**FIGURE 3.** $D_2$R-$CB_1$R-$A_{2A}$R heteromers detected by a combination of bimolecular fluorescence complementation and BRET. In (A), as a positive control, BRET saturation curve was performed using HEK-293 cells cotransfected with 0.75 µg of cDNA corresponding to the fusion protein $G_\gamma$-*Rluc* (100,000 bioluminescence units) and increasing equal amounts of cDNAs corresponding to $G_\alpha$nYFP and $G_\beta$cYFP (1000–6000 fluorescence units). In (B), confocal microscopy image of a cell after 48 h of transfection with 1 µg of cDNA corresponding to $D_2$R*Rluc*, 2 µg of cDNA corresponding to $CB_1$RnYFP, and 2 µg of cDNA corresponding to $A_{2A}$RcYFP. Proteins were identified by fluorescence (green image) or by immunocytochemistry (red image) using a monoclonal anti-*Rluc* primary antibody and a cyanine-3-conjugated secondary antibody. Colocalization is shown in yellow in the right image. In (C), BRET saturation curve (red) was obtained using HEK-293 cells cotransfected with 1.5 µg of cDNA corresponding to $D_2$R*Rluc* (100,000 bioluminescence units) and increasing equal amounts of cDNAs corresponding to $CB_1$RnYFP and $A_{2A}$RcYFP (1000–10,000 fluorescence units). As negative controls, cells were transfected with 1.5 µg of either the cDNA for $D_4$*Rluc* (black line) or for $GABAB_2$*Rluc* (green line) (100,000 bioluminescence units in each case).

There has been reported a coexpression of $D_2R$ and $A_{2A}R$ in GABAergic striatal neurons (see [9]) and of $CB_1R$ and $A_{2A}R$ in rat striatal fibrillar structures[11,13]. The demonstration of $D_2R$-$CB_1R$-$A_{2A}R$ heteromers in transfected cells, together with such striatal codistribution of the three receptors in the plasma membrane of striatal neurons, strongly suggests that these three receptors are forming part of a molecular network. The function of these neurons is particularly compromised in Parkinson's disease and in the early stages of Huntington's disease[21]. Furthermore, neuroadaptations of glutamatergic synapses of GABAergic enkephalinergic neurons localized in the nucleus accumbens (the ventral part of the striatum) seem to be involved in compulsive drug seeking and relapse[40]. Based on the existence of antagonistic interactions between $A_{2A}R$ and $D_2R$ in the $A_{2A}R$-$D_2R$ heteromer[21,25], $A_{2A}R$ antagonists are giving successful results in clinical trials in patients with Parkinson's disease[41]. Furthermore, $A_{2A}R$ antagonists are being considered as possible therapeutic agents in end-stage drug addiction[42]. Their clinical efficacy might be related to the recently demonstrated dependence of $A_{2A}R$ activation for $CB_1R$ receptor signaling within the striatal $A_{2A}R$-$CB_1R$ heteromers[11]. Thus, $A_{2A}R$ antagonists behave as $CB_1R$ antagonists, known to counteract cue-induced reinstatement of different addictive drugs in the experimental animal, a model for human relapse[43]. Although the intramembrane and intracellular cross-talk established by complexes formed by receptor heterodimers is already important to understand better the function of striatal enkephalinergic neurons, the occurrence of oligomers formed by three different receptors indicate a more diverse interplay between receptors for neurotransmitters. Taken together, the results already reported in the literature suggest that the $A_{2A}R$-$CB_1R$-$D_2R$ receptor heteromer may act as a processor mediating the neuronal computation needed to modulate striatal dopamine neurotransmission. The demonstration of $D_2R$-$CB_1R$-$A_{2A}R$ heteromers in transfected cells, together with their striatal codistribution, opens new perspectives to understand the interplay between different neurotransmitter-neuromodulator systems. Pharmacological and functional diversification expand in a macromolecular complex containing three receptors by the same simple events as described for dimers, i.e., by (1) a change in the pharmacological profile of a receptor when another receptor in the complex is activated and (2) a change in the associated signaling response-pathways depending on the receptors present in the complex, their degree of activation, and the nature of the G proteins expressed in the horizontal molecular network involved[44].

The combination of bimolecular fluorescence complementation and bioluminescence energy transfer techniques constitutes a powerful approach to detect the protein-protein interactions localized in the plane of the membrane, and thus allows identification of the horizontal molecular networks like the receptor networks in local circuits. This new knowledge will hopefully provide novel therapeutic approaches for neurodegenerative diseases, mental disorders, and drug addiction.

## ACKNOWLEDGMENTS

This research was supported by grants SAF2006–00170 and SAF2005-00170 from the Spanish Ministerio de Ciencia y Tecnologia and grant 060110 from Fundació La Marató de TV3.
